# Low-Temperature Methanolysis of Polycarbonate over Solid Base Sodium Aluminate

**DOI:** 10.1021/acs.langmuir.3c03799

**Published:** 2024-02-26

**Authors:** Philip Anggo Krisbiantoro, Miyu Sato, Tzu-Ming Lin, Yu-Chia Chang, Tzu-Yun Peng, Yun-Chih Wu, Weisheng Liao, Yuichi Kamiya, Ryoichi Otomo, Kevin C.-W. Wu

**Affiliations:** † Molecular Science and Technology Program, Taiwan International Graduate Program, Academia Sinica, Taipei 11529, Taiwan; ‡ International Graduate Program of Molecular Science and Technology, National Taiwan University, Taipei 10617, Taiwan; § Department of Chemical Engineering, College of Engineering, National Taiwan University, Taipei 10617, Taiwan; ∥ Graduate School of Environmental Science, Hokkaido University, Nishi 5, Kita 10, Kita-ku, Sapporo 060-0810, Japan; ⊥ Faculty of Environmental Earth Science, Hokkaido University, Nishi 5, Kita 10, Kita-ku, Sapporo 060-0810, Japan; # Center of Atomic Initiative for New Materials, National Taiwan University, Taipei 10617, Taiwan; ∇ Department of Chemical Engineering and Materials Science, Yuan Ze University, Chung-Li, Taoyuan 320, Taiwan

## Abstract

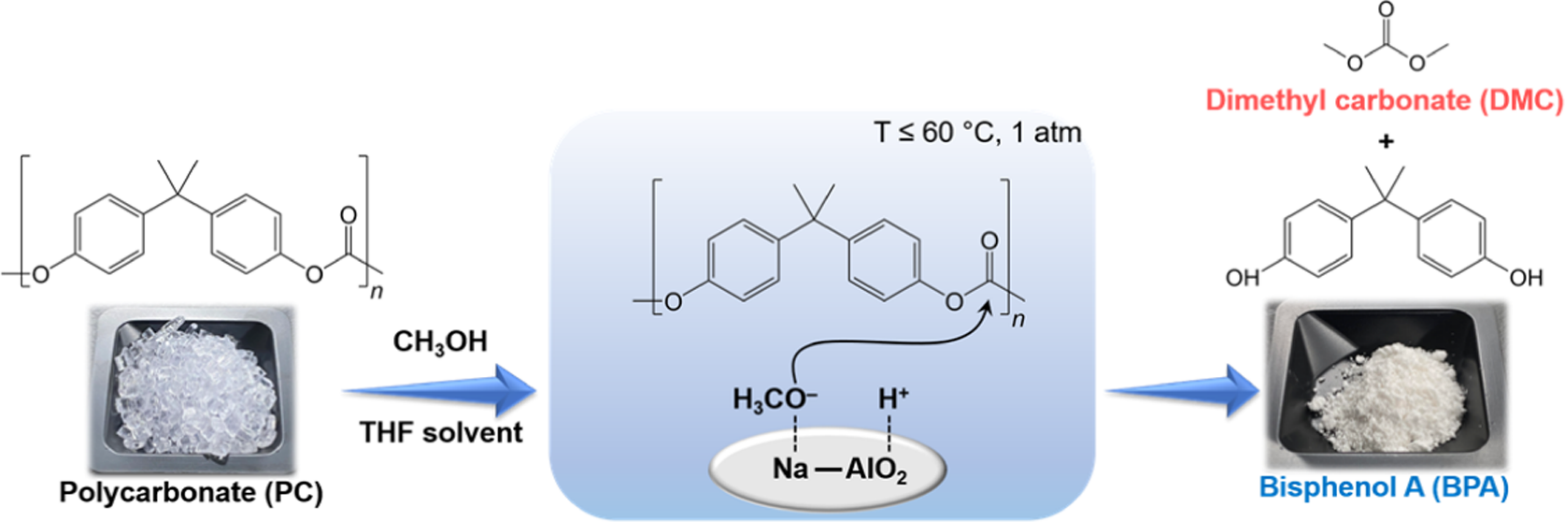

Herein, a low-cost and readily available sodium aluminate (NaAlO_2_) was used as a solid base catalyst for the depolymerization of polycarbonate (PC) via methanolysis in the presence of tetrahydrofuran (THF) as a solvent. NaAlO_2_ was highly active for the reaction, and the performance was comparable to that of soluble strong base SrO and much higher than those of MgO and CaO. By the reaction over the catalyst, a highly pure and crystalline bisphenol A (BPA) was obtained. Among tested organic solvents, THF was the best in aiding PC methanolysis over NaAlO_2_ due to the polarity similar to PC according to Hansen solubility parameters (HSPs). At 60 °C, 98.1% PC conversion and 96.8% BPA yield were achieved within just 2 h. NaAlO_2_ was reusable without any severe catalyst deactivation in at least four runs. The mechanistic study revealed that the reaction proceeded via the methoxide pathway, with THF aiding the dissolution of PC. The reaction over NaAlO_2_ possessed a low apparent activation energy (*E*_a_) of 75.1 kJ mol^–1^, which is the lowest ever reported so far for the reaction over solid catalysts.

## Introduction

Owing to the unescapable and massive daily use of nonbiodegradable plastic, plastic pollution has recently been an emerging threat to both society and the environment. Since it was first manufactured in the 1940s, about 6300 Mt of global plastic waste has been produced, and it is projected that the volume will reach 12,000 Mt by 2050.^1^ Notably, the use and disposal of polycarbonate (PC), which is one of the top engineering plastics, has increased over the years due to the fast-growing emerging industries coupled with the increased demand for biomedical devices and personal protection equipment during the COVID-19 period.^2^ Indeed, landfilled PC plastic not only leads to an inaesthetic environment but also generates micro- and nanoplastics that humans, mammals, and marine animals can easily ingest, which leads to adverse effects on their health.^3^ For instance, in the water system, bisphenol A (BPA) may leach and act as an endocrine disruptor chemical (EDC) that leads to infertility, obesity, breast and prostate cancer, and disorders in a metabolic system such as polycystic ovary syndrome (PCOS) if ingested.^3−5^ Therefore, even temporary landfilling has an adverse effect on both the environment and human health, and immediate recycling of PC plastics is indispensable.

Like other plastics, the recycling of PC is generally divided into primary and secondary routes through mechanical recycling, tertiary recycling via chemical treatments to produce monomers, and quaternary recycling to produce energy by pyrolysis.^5^ While the product from mechanical recycling possesses the drawback of values and properties often lower than those of the original products, the pyrolysis process produces toxic gases harmful to both the environment and human health. Therefore, chemical recycling has been the most promising way for PC recycling as it produces high-purity monomers that can be used for the remanufacturing of PC or epoxy resins, ensuring the attainment of completely sustainable cycles.^6^ The techniques for the chemical depolymerization of PC include alcoholysis, hydrolysis, ammonolysis, and hydrogenolysis. Principally, all of these processes allow the regeneration of BPA monomers but different coproducts, e.g., dialkyl carbonate, carbon dioxide, urea, and methanol, for alcoholysis, hydrolysis, ammonolysis, and hydrogenolysis, respectively. Among them, alcoholysis using methanol (methanolysis) has garnered much attention, as the reaction can be performed under relatively mild conditions, and the industrially important green solvent dimethyl carbonate (DMC) can be produced. However, a catalyst is necessary to increase the reaction rate, as noncatalytic PC methanolysis is a sluggish reaction.

Generally, the catalysts that can be used for PC methanolysis are base catalysts for transesterification reactions. Although many catalysts have been reported, most of them are homogeneous catalysts and there are only a few heterogeneous catalysts reported in the reaction. Those homogeneous catalysts include NaOH,^7^ organocatalyst,^8^ and ionic liquids ([Bmim][Cl],^9^ [Bmim][Ac],^10^ [Bmim]Cl·2FeCl_3_,^11^ [HDBU][LAc],^12^ and ChCl-2Urea^13^). Although these catalysts were active for the reaction, their inherent nature as a homogeneous catalyst and concentrated amount make them corrosive and difficult to separate from the medium, thus unfavorable for industrial application. Therefore, heterogeneous catalysts have recently been introduced to overcome such limitations. For instance, Zhao et al. reported that the use of SBA-15-supported CaO gave 100% PC conversion and 96% BPA yield at 130 °C for 3 h in the presence of tetrahydrofuran (THF) as a solvent.^14^ Later, Liu and co-workers doped Ca and Ce atoms into the lattice of SBA-15 through the plasma surface method to create CaO/Ce-SBA-15, a mesoporous composite with strong and abundant basic sites.^15^ Under 130 °C and 3 h of reaction time, 100% PC conversion and 94% BPA yield were obtained. The same group has then recently developed hollow CeO_2_–CaO–ZrO_2_ and 100% PC conversion and ca. 96% BPA yield was achieved at 100 °C and 2 h, which were lower and shorter than in their previous work.^16^ Although these catalysts were recyclable, they are complex composites that require careful and tedious preparation. In addition, the reaction temperature was still higher than the boiling point of methanol (ca. 64.7 °C), and consequently, the reaction must be carried out in a closed system such as an autoclave. It is worth mentioning that a heterogeneous catalytic system for PC methanolysis under atmospheric pressure and at temperatures lower than the boiling point of methanol has not yet been reported so far. Therefore, a simple and low-cost heterogeneous catalyst active for the reaction even under atmospheric pressure, which is satisfactory in the industrial field, is keenly desired.

Sodium aluminate is an inorganic chemical with a general formula NaAlO_2_ that has numerous applications. NaAlO_2_ is generally used as a coagulant in municipal drinking water and wastewater treatment processes, while it has also been widely used in construction technology, paper and paint industries, zeolite synthesis, and so on.^17^ Despite its versatile applications, NaAlO_2_ has rarely been applied as a heterogeneous catalyst, and only a few have realized the promising potential of this material as a low-cost solid base catalyst, with most of the reports being transesterification reactions.^18−23^ For instance, Debecker and co-workers reported that NaAlO_2_ is an excellent catalyst (more active than solid base CaO and SrO) for the transesterification of sunflower oil with methanol to produce fatty acid methyl esters (FAME) with over 90% yield under 60 °C for 4 h.^23^ As PC methanolysis is also a reaction promoted by a base catalyst, NaAlO_2_ is a promising heterogeneous base catalyst for the reaction because it is insoluble in methanol.^18,23^ To the best of our knowledge, the use of NaAlO_2_ for PC methanolysis has not yet been reported so far. Therefore, in this work, NaAlO_2_ was tested for its catalytic activity for the reaction under atmospheric pressure, at temperatures lower than the boiling point of methanol, and in the presence of THF as a solvent. While the catalytic performance of NaAlO_2_ was first compared with common solid base catalysts, e.g., MgO, CaO, and SrO, at room temperature, the effects of solvent, kinetics, and plausible mechanism for the reaction were investigated. Under optimized conditions, the reusability was also evaluated.

## Experimental Section

### Materials

PC pellets (2.0 × 3.0 × 3.0 mm^3^) were obtained from a local supplier in Taiwan. All chemicals were of analytical grade and used without further purification. Acetonitrile (99.9% w/w), acetone (≥99.5%), heptane (≥99%), and methanol (anhydrous, 99%) were obtained from Macron. Chloroform (≥99% w/w) was purchased from Honeywell. While dichloromethane (99%), deuterated dimethyl sulfoxide (DMSO-*d*_6_, 99.9% atom D), magnesium oxide (97%), 4-nitroaniline (≥99%), sodium aluminate, sodium methoxide (25wt % in methanol), and sodium chloride were provided by Sigma-Aldrich, cyclohexane (≥99.0%) was acquired from J.T. Baker. Alizarin yellow R sodium salt, calcium oxide (99.95%), phenolphthalein, and strontium oxide (99.5%) were acquired from Alfa Aesar. Bisphenol A (97%), bromothymol blue, dimethyl carbonate (99%), and tetrahydrofuran (99.6%) were purchased from Thermo Fisher Scientific. Meanwhile, octane (>98%) was obtained from Wako.

### General Procedure for PC Methanolysis

In a typical reaction, 5 g of methanol (MeOH) and 10 g of THF were placed in a 50 mL round-bottom flask equipped with a magnetic stirrer (for a reaction at room temperature) or a three-neck flask equipped with a thermometer, condenser, and magnetic stirrer (for a reaction at *T* ≥ 40 °C). The flask was then placed in a magnetic stirrer or a magnetic stirrer equipped with an electric heater. For the reaction at room temperature, 5 g of PC pellets were added into the flask, which was quickly followed by the addition of 0.2 g of catalyst. As for the reaction at *T* ≥ 40 °C, both PC and catalyst were added after the reaction solution reached the predetermined temperature. It should be noted that all tested catalysts were lyophilized overnight before being used for the reaction to remove the moisture. The mixture was then vigorously stirred for a predetermined amount of time under atmospheric pressure. After the reaction, the catalyst and unreacted PC were separated by filtration. After the PC residue was separated from the catalyst and dried at 60 °C for 24 h, it was weighed to calculate the PC conversion according to eq 1,
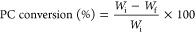
1where *W*_i_ and *W*_f_ are the weights of the initial PC dose and the PC residue, respectively. Meanwhile, to separate the oligomers, 30–50 mL of MeOH was added to the filtrate. After being left to stand overnight, the as-formed white precipitate was separated by filtration. The filtrate was then evaporated by a rotary evaporator into dryness to obtain pure BPA. The formed solid was then further dried in a lyophilizer overnight, and the yield of BPA can be calculated according to eq 2,
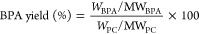
2where *W*_PC_ and *W*_BPA_ are the weight of PC dose and obtained BPA, respectively, while MW_PC_ and MW_BPA_ correspond to molar masses of one repeating unit of PC (254 g mol^–1^) and BPA (228 g mol^–1^), respectively.

### Characterization

Powder X-ray diffraction (XRD) patterns of catalyst and BPA were recorded on a powder X-ray diffractometer (SmartLab SE, Rigaku), while the surface of the PC samples was observed by a scanning electron microscope (Hitachi S-4800). The specific surface area of the catalyst was estimated by applying the Brunauer–Emmett–Teller (BET) theory to a N_2_ adsorption–desorption isotherm, which was taken on a BELSORP-Max II nitrogen analyzer. While the functional groups of the as-produced BPA were characterized by using a Fourier-transform infrared spectrometer (PerkinElmer Spectrum), its purity was identified using differential scanning calorimetry/thermogravimetry (TA Instruments, SDT 650) and ^1^H NMR (Bruker, drx500). Meanwhile, the generated DMC was analyzed by using a gas chromatograph-flame ionization detector (Shimadzu, GC-2025) with a ZB-1 capillary column (30 m × 25 mm × 50 μm) and octane as an internal standard.

## Results and Discussion

### Catalytic Performance of NaAlO_2_

**Figure 1:**
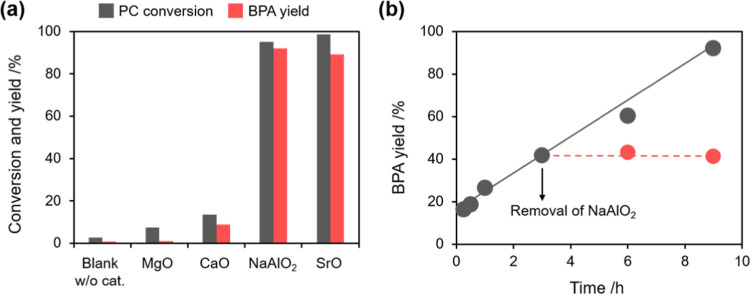
(a) Catalytic performance of NaAlO_2_ in comparison to alkaline-earth metal oxide catalysts, e.g., CaO, MgO, and SrO for PC methanolysis in the presence of THF as a solvent (reaction conditions: catalyst, 0.2 g; MeOH, 5 g; THF, 10 g; PC, 5 g; reaction temperature, room temperature; and reaction time, 9 h). (b) The filtration test for PC methanolysis in the presence of THF over NaAlO_2_ with and without the removal of NaAlO_2_ at 3 h (reaction conditions: catalyst, 0.2 g; MeOH, 5 g; THF, 10 g; PC, 5 g; reaction temperature, room temperature; and reaction time, 9 h).

Figure 1a shows the catalytic performance of commercial NaAlO_2_ with the catalytic reaction data for commercially available solid base catalysts, e.g., CaO, MgO, and SrO. Under a MeOH/THF/PC weight ratio of 1:2:1, NaAlO_2_ exhibited high catalytic performance (95% PC conversion and 92% BPA yield) comparable to the strong base SrO (98% PC conversion and 89% BPA yield) at room temperature for 9 h. While CaO was the second-best alkaline-earth metal oxide for the reaction (13.6% PC conversion and 8.9% BPA yield), MgO was the least active as it only gave 7.5% PC conversion and negligible BPA yield. It is noted that although SrO exhibited the highest catalytic performance, part of it was dissolved under the reaction conditions (more than 40% of the weight was lost during the reaction). In addition, the XRD pattern of SrO was completely changed during the reaction, and it became amorphous after the reaction, implying the destruction of the crystal structure during PC methanolysis (Figure S1a). Therefore, SrO was not suitable as a solid base catalyst for the reaction. This is in contrast with NaAlO_2_, where, despite being a salt, the solubility in the reaction solution was negligible, and the crystal structure after the reaction was identical to that before the reaction (Figure S1b).

To understand the parameter governing the catalytic activity among the tested catalysts, base strength and amount of the basic site of the catalysts were estimated by titration with Hammett indicators, and the results are summarized in Table 1. To determine the base strength, four Hammett indicators, including bromothymol blue (*H*_–_ = 7.2), phenolphthalein (*H*_–_ = 9.8), alizarin yellow R (*H*_–_ = 11.0), and 4-nitroaniline (*H*_–_ = 18.4), were used with MeOH as a solvent (the detailed experimental procedure can be seen in Figure S2). The experiments revealed that the base strength of MgO and CaO was 7.2 ≤ *H*_*–*_ ≤ 9.8, NaAlO_2_ and SrO exhibited stronger base strength, i.e., 9.8 ≤ *H*_*–*_ ≤ 11.0 and 11.0 ≤ *H*_*–*_ ≤ 18.4, respectively (Table 1). These facts can explain the difference in the catalytic activities; while MgO and CaO exhibited low catalytic performance, SrO and NaAlO_2_ showed otherwise (Figure 1a), implying that the difference in the catalytic activity was due to the difference in the base strength of the catalyst. Later, a titration experiment was performed to quantify further the amount of basic site of each catalyst with bromothymol blue as an indicator, MeOH as a solvent, and HCl as a titrant (the detailed experimental procedure is shown in the Supporting Information). As shown in Table 1, the order of the number of basic sites was SrO (6.18 mmol g^–1^) > NaAlO_2_ (0.90 mmol g^–1^) > CaO (0.15 mmol g^–1^) > MgO (0.06 mmol g^–1^), which matched the order of the catalytic performances shown in Figure 1a. Overall, these results clearly suggest that the large number of basic sites of NaAlO_2_ is the reason for the high catalytic performance. It is worth noting that the strong basic sites of NaAlO_2_ have also been reported to be the ones responsible for the high catalytic activity of some esterification reactions.^18−22^

**Table 1:** Chemical and Physical Properties of the As-Tested Catalysts

catalyst	*S*_BET_, m^2^ g^–1^	*H*_*–*_	amount of basic site, mmol g^–1^
MgO	5.6	7.2 ≤ *H*_*–*_ ≤ 9.8	0.06
CaO	3.0	7.2 ≤ *H*_*–*_ ≤ 9.8	0.15
NaAlO_2_	0.75	9.8 ≤ *H*_*–*_ ≤ 11.0	0.90
SrO	1.5	11.0 ≤ *H*_*–*_ ≤ 18.4	6.18

To check the contribution of the species leached from NaAlO_2_ during the reaction, a filtration test was performed (the detailed experimental procedure is seen in the Supporting Information). As shown in Figure 1b, the BPA yield increased with reaction time and reached 99% at 9 h in the presence of NaAlO_2_ over the reaction time. On the other hand, the BPA yield did not increase after the catalyst was removed from the reaction solution at 3 h. This result clearly demonstrates that the contribution of the dissolved species to the reaction was almost negligible, even if it existed.

**Figure 2:**
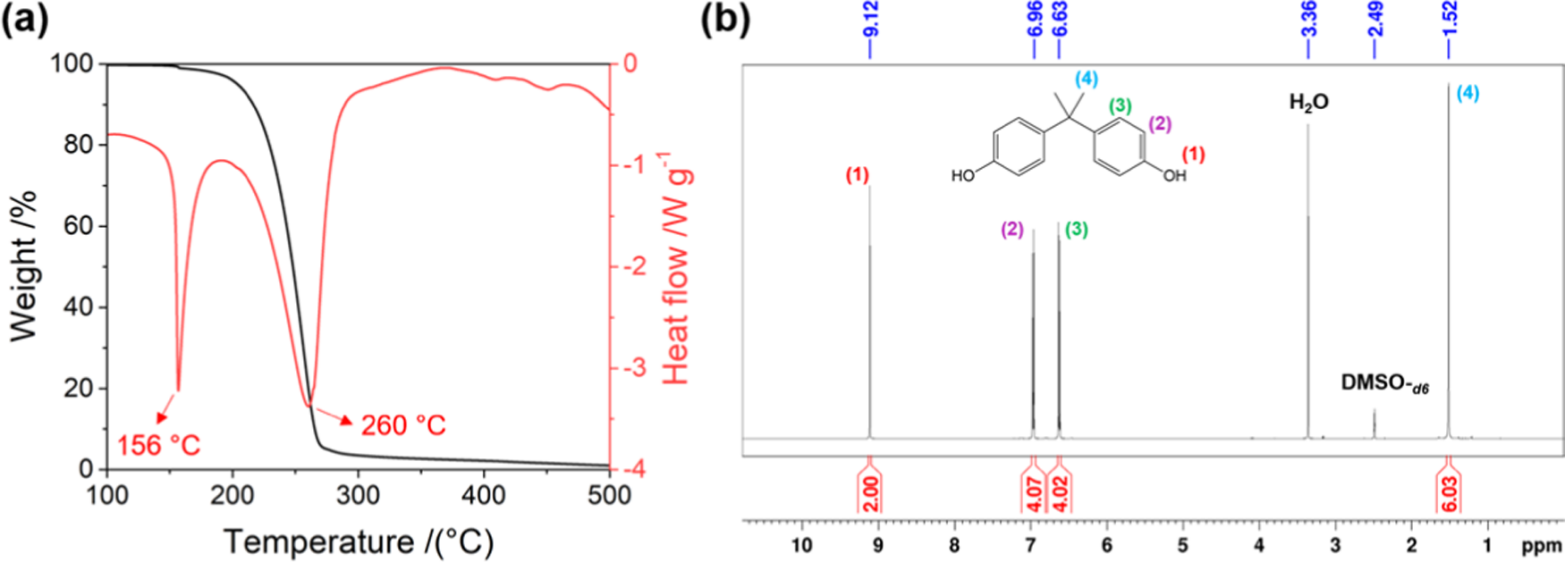
(a) TGA-DSC curve and (b) ^1^H NMR patterns of as-produced BPA from PC methanolysis over NaAlO_2_.

To further confirm that the depolymerization product formed by PC methanolysis was indeed BPA, the product obtained by the reaction with NaAlO_2_ was characterized with XRD, FTIR, TGA, and ^1^H NMR. The obtained BPA exhibited an XRD pattern and FTIR spectrum identical to that of commercial BPA (Figure S3). TGA-DSC analysis showed that only one endothermic peak attributed to the melting point of BPA at 156 °C was observed for the obtained BPA, which implies the absence of a dimer, trimer, or oligomer (Figure 2a). The peak at 260 °C, on the other hand, can be assigned to the decomposition of BPA. On the ^1^H NMR spectrum of the obtained BPA (with DMSO-*d*_6_ as a solvent) (Figure 2b), a singlet peak at 9.12 ppm characteristic of the phenolic hydroxyl group was observed, and the doublet peaks at 6.96 and 6.63 ppm were the typical ones due to the protons at *ortho* and *meta* positions of the benzene ring, respectively. Meanwhile, the singlet peak at 1.52 ppm was the characteristic chemical shift of the methyl group in BPA. Overall, these characterization results demonstrate that the product obtained by PC methanolysis over NaAlO_2_ was indeed highly pure BPA.

### Organic Solvent-Assisted PC Methanolysis

As PC methanolysis is a sluggish reaction, a reaction temperature higher than the boiling point of MeOH is generally applied to obtain a practical depolymerization rate. Consequently, a closed system is required, as high pressure is generated during the reaction. To achieve a lower reaction temperature, an organic solvent can be added to assist the dissolution of PC for a higher methanolysis rate. Although it has been reported that the addition of toluene enables PC methanolysis at 40 °C with NaOH as a catalyst,^7^ no solvent-aided heterogeneous catalysis has achieved a reaction temperature lower than the boiling point of MeOH. For instance, the use of ZnO-supported ionic liquid (ZnO-NPs/NBu_4_Cl)^24^ and CaO/Ce-SBA-15^15^ composite in the presence of THF as a solvent only allows the reaction to be done at 100 and 130 °C, respectively. As we are the first group to report low-temperature PC methanolysis in the presence of solid NaAlO_2_ with the aid of THF, it is important to investigate the effect of solvent on the catalytic performance of the system. Therefore, in this work, eight organic solvents with different polarities, e.g., acetone, acetonitrile (ACN), chloroform, cyclohexane, dichloromethane (DCM), dimethyl carbonate (DMC), heptane, and THF, were tested for their effect on the PC conversion and BPA yield for the reaction over NaAlO_2_ at room temperature.

**Figure 3:**
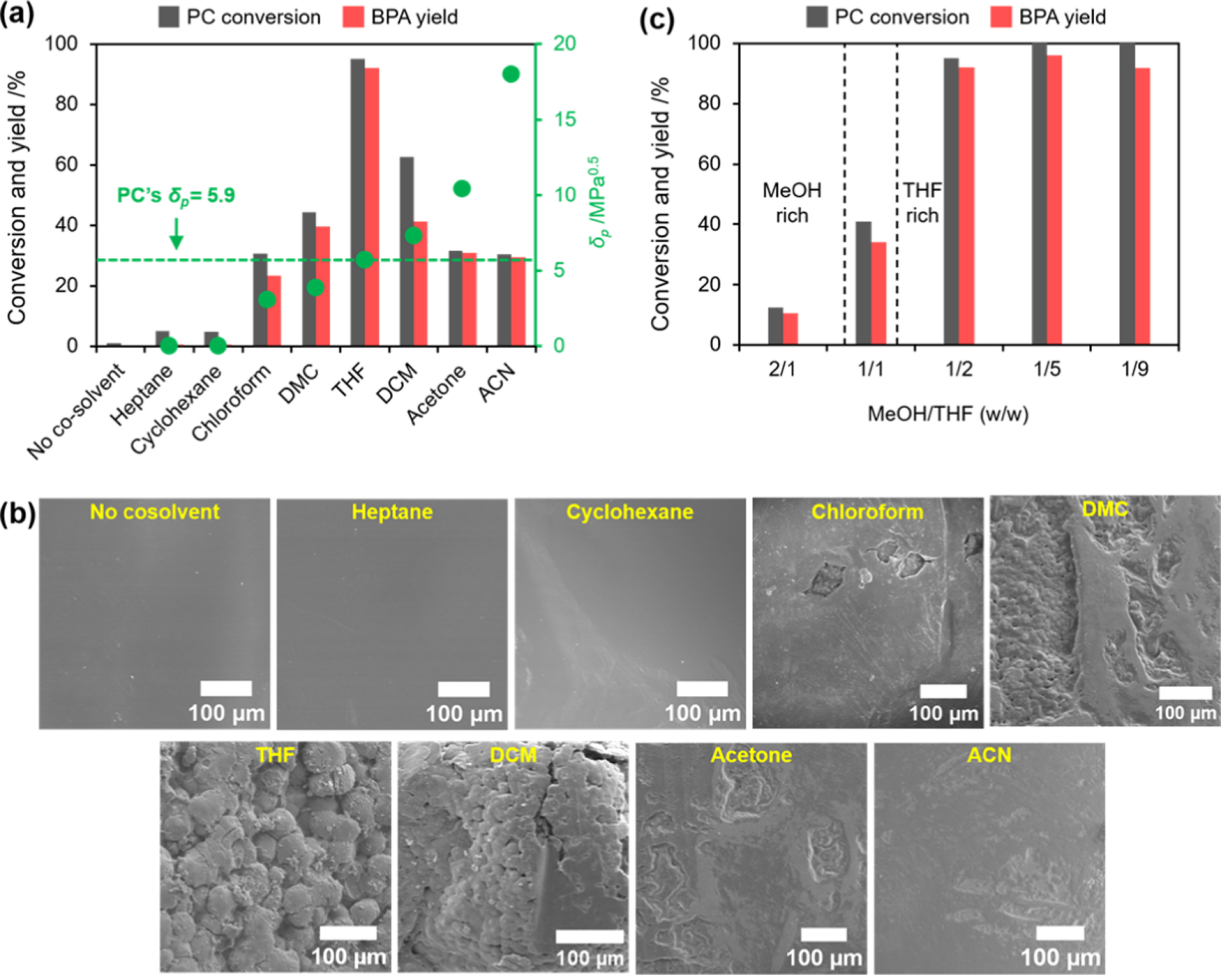
(a) Catalytic performance of NaAlO_2_ for PC methanolysis in the presence of different organic solvents (reaction conditions: catalyst, 0.2 g; MeOH, 5 g; solvent, 10 g; PC, 5 g; reaction temperature, room temperature; reaction time, 9 h). (b) SEM images of PC pellets treated with different MeOH/solvents (treatment conditions: MeOH, 1 g; solvent, 2 g; PC, 1 g; treatment temperature, room temperature; stirring time, 2 h). (c) The effect of the MeOH/THF ratio on the PC methanolysis over NaAlO_2_ (reaction conditions: catalyst, 0.2 g; MeOH and THF, 15 g; reaction temperature, room temperature; and reaction time, 9 h).

Figure 3a displays the catalytic performance of NaAlO_2_ in the presence of organic solvents sorted from left to right based on their polarity, while Table 2 summarizes the Hansen solubility parameters (HSPs), e.g., dispersion (δ_d_), polar (δ_p_) and hydrogen-bonding (δ_h_) forces, of MeOH, PC, and solvents along with their *R*_a_ index to MeOH and PC. The *R*_a_ index was calculated by using the formula (*R*_a_)^2^ = 4(δ_d,1_ – δ_d,2_)^2^ + (δ_p,1_ – δ_p,2_)^2^ + (δ_h,1_ – δ_h,2_)^2^, where δ_d,1_, δ_p,1_, and δ_h,1_ represent the HSP values of molecule 1, while δ_d,2_, δ_p,2_, and δ_h,2_ represent the HSP values of molecule 2.^25,26^ It is noted that HSPs are used in this work to understand the difference in the catalytic performance among solvents, as it has been widely reported that HSPs are a good tool for understanding the solubility of polymers in organic solvents. While it seems that there is no correlation between the *R*_a_ index of solvent to MeOH, higher PC conversion and BPA yield was observed on the organic solvent exhibiting a smaller *R*_a_ index and closer polar parameter (δ_p_) to PC (Figure 3a and Table 2). Although the trend generally shows that the higher catalytic activity is achieved with the organic solvent possessing a smaller *R*_a_ index to PC, chloroform (*R*_a_ to PC = 3.1) did not follow the trend as it delivered low performance despite having a low *R*_a_ index to PC. Similarly, DCM (*R*_a_ to PC = 2.8) also gave much lower performance than THF (*R*_a_ to PC = 3.0), although it has a smaller *R*_*a*_ index to PC than THF (Figure 3a and Table 2). Therefore, in this work, δ_p_ was used instead of the *R*_a_ index to understand the difference in the catalytic activity among organic solvents, as it shows a better trend (Figure 3a). As clearly depicted, both PC conversion and BPA yield increased with the increase in the polar parameter (δ_p_) of the solvent, i.e., from heptane to THF, but then decreased with the further increase in the polarity of the solvent (Figure 3a). This can be explained as follows. In the case of heptane and cyclohexane, where only 5% PC conversion with negligible BPA yield (<1%) was observed, the big difference in the polarity between them to MeOH and PC is the primary reason for the deficient activity. According to HSPs in Table 2, δ_p_ values of MeOH and PC are 12.3 and 5.9 MPa^0.5^, respectively, while those of heptane and cyclohexane are both 0.0 MPa^0.5^. This significant difference results in neither solvent mixing with highly polar MeOH, which consequently hinders the methanolysis reaction. In the case of chloroform, although it is a nonpolar solvent, it has the δ_p_ (3.1 MPa^0.5^) value close to that of PC (5.9 MPa^0.5^), thus being able to aid the dissolution of PC. As a result, the PC conversion and BPA yield with chloroform as a solvent were higher than those with heptane and cyclohexane. Both PC conversion and BPA yield further increased when DMC and THF were used as solvents, with the performance in the THF solvent being higher than the one in the DMC solvent as the δ_p_ value of THF (5.7 MPa^0.5^) is closer to PC than DMC (3.9 MPa^0.5^). When more polar solvents, e.g., DCM (δ_p_ = 7.3 MPa^0.5^), acetone (δ_p_ = 10.4 MPa^0.5^), and ACN (δ_p_ = 18.0 MPa^0.5^) were used, both PC conversion and BPA yield decreased but were still significantly higher than those with nonpolar solvents. This clearly indicates that if the solvents become too polar, the affinity to PC decreases as PC has nonpolar benzene rings and methyl groups within its polymeric chains. However, as these solvents are miscible with MeOH, they gave better performance than those in nonpolar ones.

**Table 2:** Hansen Solubility Parameters of Organic Solvents Used in this Work and their *R*_a_ Index from MeOH and PC

solvent	δ_d_, (MPa)^0.5^	δ_p_, (MPa)^0.5^	δ_h_, (MPa)^0.5^	*R*_a_ from MeOH	*R*_a_ from PC
heptane	15.3	0.0	0.0	25.5	10.8
cyclohexane	16.8	0.0	0.2	25.6	9.4
chloroform	17.8	3.1	5.7	20.0	3.1
DMC	15.5	3.9	9.7	15.2	6.4
THF	16.8	5.7	8.0	16.3	3.0
DCM	17.0	7.3	7.1	16.6	2.8
acetone	15.5	10.4	7.0	15.5	7.0
ACN	15.3	18.0	6.1	17.2	13.4
MeOH	14.7	12.3	22.3		
PC	18.2	5.9	6.9		

To gain insight into the influence of solvents on the morphology change of PC, the SEM images of PC pellets after 2 h treatment in the mixture of MeOH and solvent (MeOH/solvent = 1/2) without catalysts were taken and compared to the one treated in only MeOH without any solvent (Figure 3b). While nonpolar solvents, e.g., heptane and cyclohexane, did not cause a change in morphology, the use of chloroform resulted in the formation of small holes and a slightly rough surface. Meanwhile, obvious cracks with rough surfaces were observed in DMC, THF, and DCM, with THF being the most apparent one. In the case of THF, not only was it the roughest in the surface morphology, but it also had more exposed cracks. This clearly demonstrates that THF had the highest ability to create surface roughness and cracks essential for facilitating the diffusion of MeOH and the fine powder of the catalyst into the polymer matrix.

As THF was the best solvent for PC methanolysis over NaAlO_2_, the effect of the MeOH/THF ratio was investigated. As shown in Figure 3c, the catalytic performance increased with the increase of the THF amount, i.e., the MeOH/THF ratio up to 1/2. This increase in the performance with the THF amount is because the higher the amount of THF, the faster the swelling and dissolution of PC as compared to that of a smaller amount, leading to an easier diffusion of fine powder of the catalyst and MeOH to the polymeric chain of PC.^7^ As the further increase in the amount of THF did not significantly increase the catalytic performance, we considered that a MeOH/THF ratio of 1/2 was the optimum and was applied for further investigations.

### Effect of Catalyst Weight and Temperature

**Figure 4:**
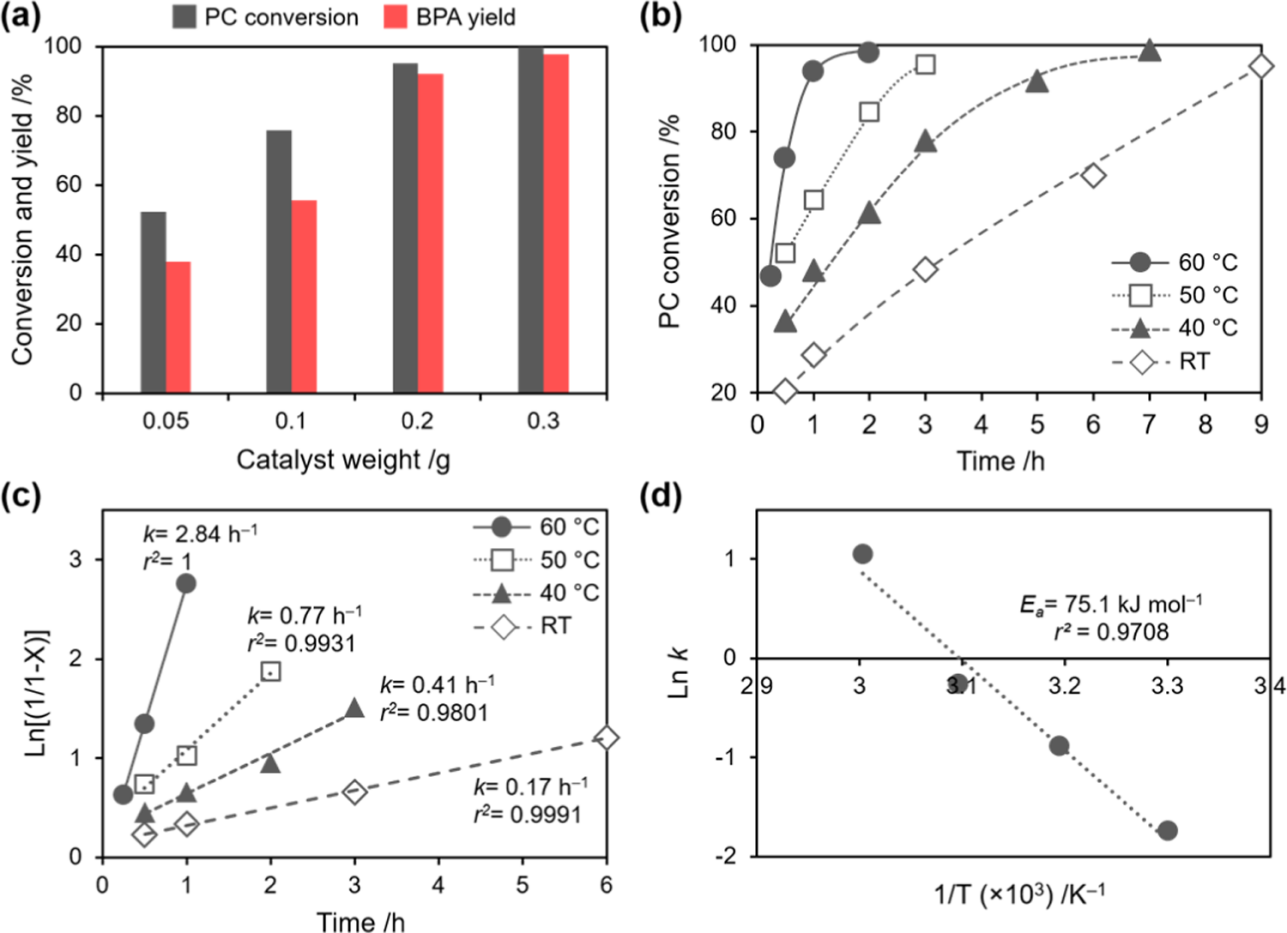
(a) Effect of the amount of catalyst (reaction conditions: MeOH, 5 g; THF, 10 g, PC, 5 g; reaction temperature, room temperature; and reaction time, 9 h), (b) PC conversion at different temperatures (reaction conditions: catalyst, 0.2 g; MeOH, 5 g; THF, 10 g; PC, 5 g), (c) the linear plot of the first-order kinetic model at different temperatures, and (d) the Arrhenius plot of the rate constant of PC methanolysis.

Here, the catalyst weight was optimized prior to the investigation of the effect of the temperature on the methanolysis rate. When 0.05 g of catalyst was added, 52.2% PC conversion and 38.0% BPA yield were obtained (Figure 4a). Both conversion and yield increased with the added amount of catalyst and then almost reached complete conversion when 0.2 g of catalyst was used, where 95.1% PC conversion and 92.1% BPA yield were achieved. This steady increase in the performance with the catalyst weight is because the higher the amount of catalyst, the larger the surface area of the active sites as compared to that of a smaller amount, leading to a further increase in the methanolysis rate of PC. Therefore, in this work, 0.2 g was chosen as the amount of catalyst used for the subsequent investigation.

Next, the effect of the reaction temperature was investigated under the optimum MeOH/THF ratio and catalyst weight to understand the catalytic performance of NaAlO_2_. As shown in Figure 4b, the reaction temperature significantly affected the reaction rate for PC methanolysis; i.e., the equilibrium state was reached faster in higher temperatures. At room temperature, the equilibrium state could only be reached after 9 h, while it can be reached at around 5, 3, and 1 h at 40, 50, and 60 °C, respectively. Notably, at 60 °C, 98% or more of PC conversion can be achieved in just 2 h with 96.8% BPA yield. Under this condition, ca. 75% DMC yield was obtained from the reaction. It is noted that the reaction at 60 °C or higher should be avoided to prevent the loss of both MeOH and THF due to evaporation.

To estimate the rate constant (*k*) for PC methanolysis at different reaction temperatures, we applied a first-order kinetic model. It should be noted that this kinetic model is typically used for PC methanolysis as an excess amount of MeOH is used for the reaction.^7,10,11,13^ The first-order rate constant (*k*) can be estimated by plotting  as a function of *t*, where *X*, *k*, and *t* are the PC conversion, first-order rate constant (h^–1^), and reaction time, respectively. As depicted in Figure 4c, a high correlation coefficient, i.e., >0.98, was obtained for all reaction temperatures, demonstrating that the first-order kinetic model was plausible. From the slope of the plot, the first-order rate constants were estimated to be 2.84, 0.77, 0.41, and 0.17 h^–1^ for the reaction at 60, 50, and 40 °C and room temperature, respectively. The apparent activation energy (*E*_*a*_) for the reaction over NaAlO_2_ can then be estimated by applying the Arrhenius equation, i.e., , to the rate constant for PC methanolysis (Figure 4d). It was revealed that PC methanolysis over NaAlO_2_ exhibited an *E*_a_ of 75.1 kJ mol^–1^. This value is significantly lower than those found in the previously reported heterogeneous catalysts, e.g., CaO-Al_2_O_3_ (145.5 kJ mol^–1^) and CeO_2_–CaO–ZrO_2_ (109.3 kJ mol^–1^), which also employed THF as a solvent,^16^ demonstrating a distinct efficiency of NaAlO_2_ as a heterogeneous catalyst. This stark difference, regardless of using the same solvent, might be caused by the fact that the basic strength of NaAlO_2_ is stronger than CaO in CaO–Al_2_O_3_ and CeO_2_–CaO–ZrO_2_, rendering a faster activation of methanol into methoxide anion during the reaction.

### Plausible Reaction Mechanism

**Figure 5:**
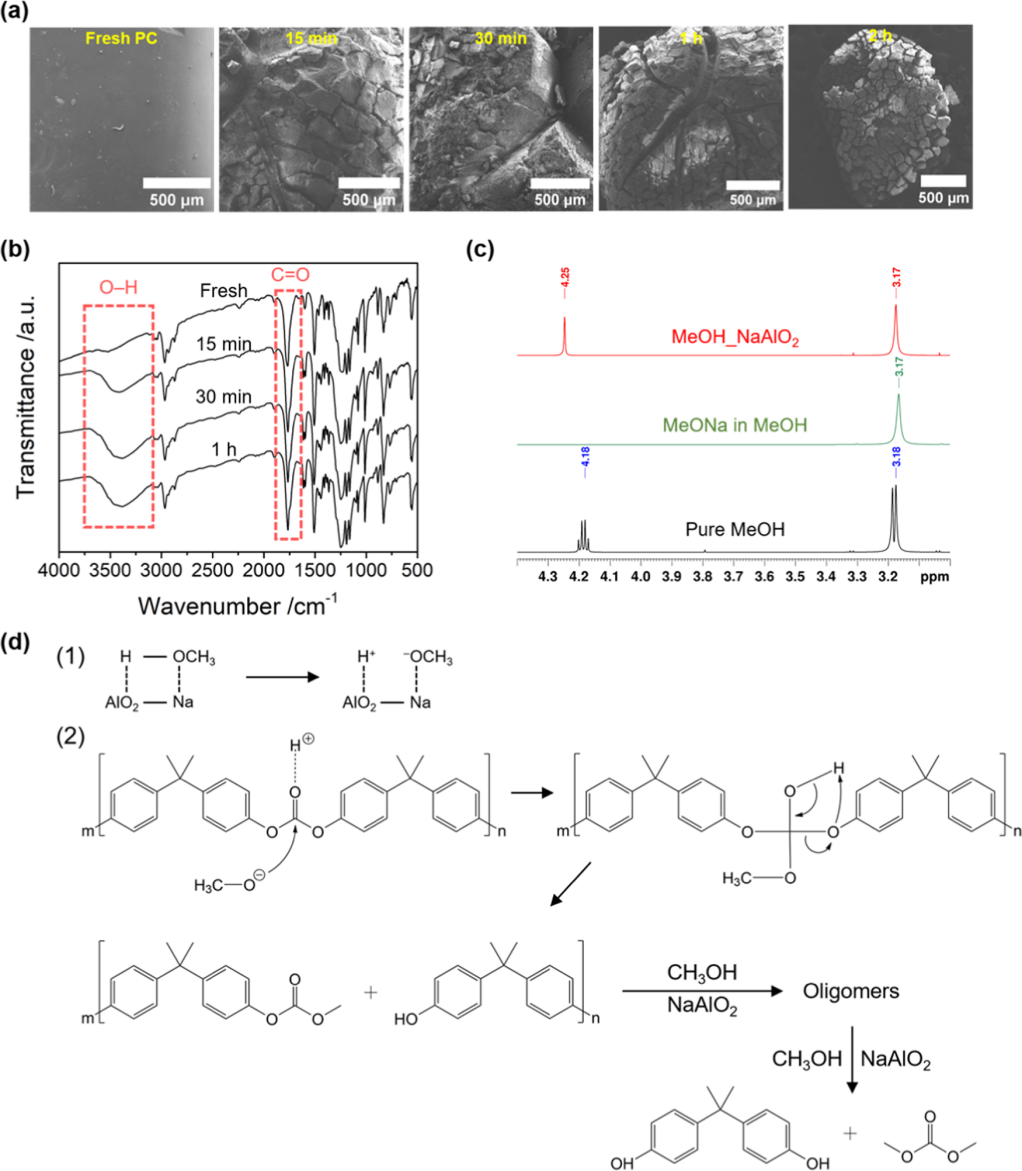
(a) SEM images of PC before and after different reaction times, e.g., 0.25, 0.5, 1, and 2 h, (b) FTIR spectra of the PC residue from the reaction at 60 °C and different reaction times in comparison to fresh PC, (c) ^1^H NMR of MeOH treated with NaAlO_2_ in the presence of NaCl in comparison with MeONa and pure MeOH, and (d) the plausible reaction mechanism for PC methanolysis over NaAlO_2_.

As it is accepted that the reaction for depolymerization of plastic occurs on the surfaces of plastic, it is important to first study the surface morphology of PC pellets during the reaction. Therefore, in this work, SEM images of PC before and after the reactions with different reaction times at 60 °C over NaAlO_2_ were taken to gain a clear understanding of the surface morphology during catalytic methanolysis. As Figure 5a clearly shows, while fresh PC had a smooth surface, the surface became very rough with large cracks within 15 min. This indicates that although the PC methanolysis began from the surface, the penetration of MeOH aided by THF occurred rapidly. The surface roughness was observed to increase with the reaction time, leaving only a tiny chunk of PC after a 2 h reaction. To further gain insight into the chemistry of the surface of PC, the PC residues obtained by the reaction at 60 °C with different reaction times were characterized by using FTIR. As shown in Figure 5b, PC residue showed peaks similar to that of fresh PC with an increase in the intensity of both −OH and −C=O at ca. 3500 and 1760 cm^–1^, respectively, with the increase in reaction time. This clearly suggests the breakage of the PC polymer into a shorter polymer chain, leading to the exposure of more −OH and −OCOOCH_3_ functional groups. Therefore, it is plausible that the PC was converted into oligomers before being finally depolymerized into BPA monomers. Similar results have also been reported previously.^10,27^

Since NaAlO_2_ is a solid base material, the presence of NaAlO_2_ in the reaction solution strongly affects the local environment of the proton from the hydroxyl group of MeOH. To determine whether MeOH was converted into a methoxide anion or remained unchanged in the presence of NaAlO_2_, ^1^H NMR analysis was conducted on MeOH after being treated with NaAlO_2_ in the presence of NaCl (denoted as MeOH_NaAlO_2_) and compared to pure MeOH and commercial sodium methoxide (MeONa) (Figure 5c). The detailed experiment can be seen in the Supporting Information. It should be noted that NaCl was added as a countercation in the case of methoxide anion formed by this treatment. As clearly depicted, the characteristic peaks for pure MeOH, i.e., doublet and quartet peaks at 3.18 and 4.18 ppm, respectively, underwent significant changes after treatment with NaAlO_2_ in the presence of NaCl. The doublet peak of the methyl group became a singlet peak and slightly shielded to 3.17 ppm, indicating that it has no neighboring proton. This chemical shift was the same as that of the methyl group observed for MeONa in MeOH, indicating the formation of a methoxide anion in MeOH with NaAlO_2_. Another peak was also observed as a singlet at a more deshielded chemical shift of 4.25 ppm, further supporting the formation of a methoxide anion.

Based on the results mentioned so far, the reaction mechanism for PC methanolysis over NaAlO_2_ can be proposed as follows. First, PC dissolves or swells in the presence of THF, which leads to the creation of cracks and facilitates the smooth diffusion of fine powder of the catalyst and MeOH to the polymer matrix.^7^ Concomitantly, the basic site of NaAlO_2_ activates MeOH to form a surface methoxide anion (Figure 5d(1)), which subsequently attacks the C=O of the carbonate group in PC, resulting in the breakdown of the polymer chain to produce oligomers and, eventually, BPA and DMC (Figure 5d(2)). The ability of methoxide anion to depolymerize PC is supported by the fact that 100% PC conversion was achieved when a very small amount of NaOMe, i.e., 0.3 wt % of NaOMe in MeOH, was used for the reaction under optimum conditions in the absence of NaAlO_2_.

### Reusability Test

**Figure 6:**
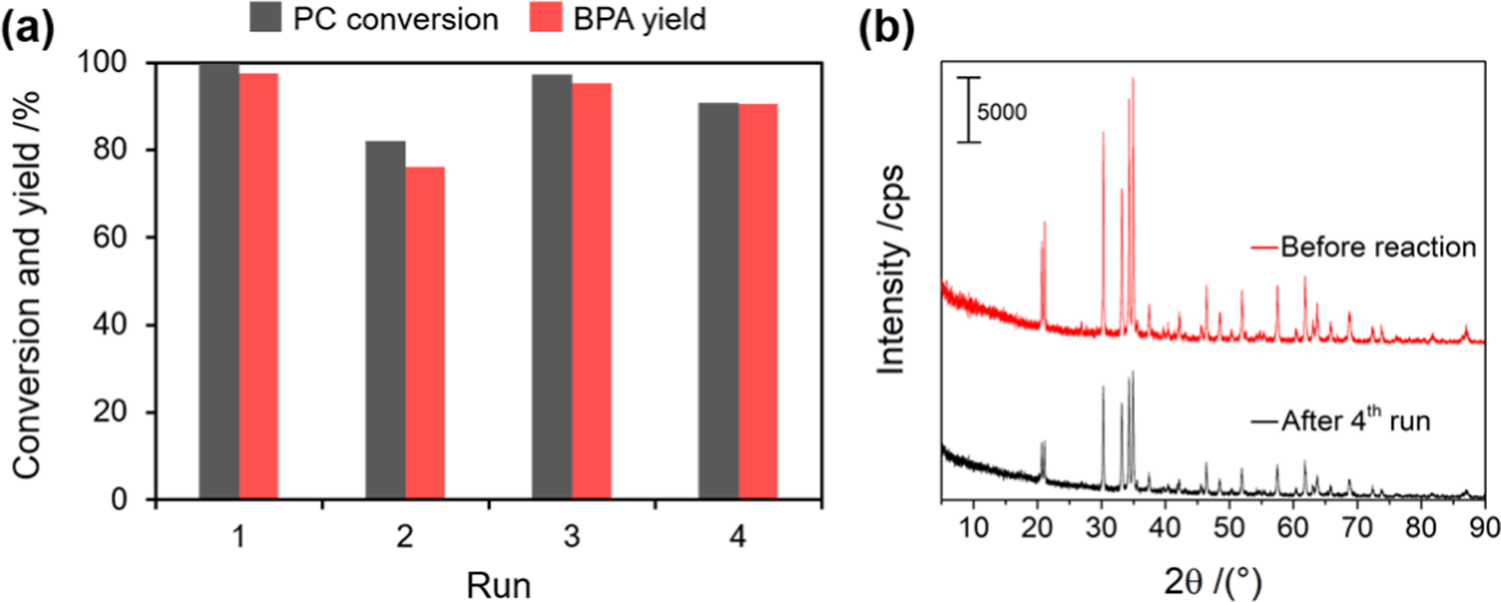
(a) Reusability test of NaAlO_2_ and (b) XRD patterns of NaAlO_2_ before the reaction and after the fourth run. Reaction conditions: catalyst, 0.2 g; MeOH, 5 g; THF, 10 g; PC, 5 g; reaction temperature, 60 °C; and reaction time, 2 h.

Under optimum conditions, the reusability of NaAlO_2_ was investigated to demonstrate the durability of the catalyst for PC methanolysis (the detailed experimental procedure is seen in the Supporting Information). As shown in Figure 6a, the catalyst can be reused at least 4 times. While the decrease in the catalytic activity on the second reuse could be caused by the poisoning of the catalyst surface by BPA or oligomers, the increase in the performance on the third and fourth reuses might be caused by the increase in the dispersion of the catalyst with the cycling time, which overwhelms the deactivated active sites. The decrease in the catalytic activity of NaAlO_2_ has been observed in other reactions.^18,22,23^ Meanwhile, the fact that there was no change in the diffraction patterns due to NaAlO_2_ before and after being used four times in a row suggests that the catalyst was stable (Figure 6b). Therefore, it is concluded that NaAlO_2_ is a reusable and highly active solid catalyst for PC methanolysis in the presence of THF as a solvent.

## Conclusions

In summary, NaAlO_2_ is an active catalyst for PC methanolysis in the presence of tetrahydrofuran (THF) as a solvent with the high basicity of the catalyst as the primary reason for the high catalytic performance. Among tested organic solvents, THF was the best solvent for aiding the reaction, as according to HSPs, it has the closest δ_p_ value to that of PC, thus increasing the affinity between the two and leading to easy dissolution. Not only was high purity of BPA obtained, but the reaction can be done under room temperature, i.e., 95% PC conversion and 92% BPA yield, in 9 h. The optimum conditions were achieved at 60 °C and 2 h reaction time, where 98.1% PC conversion and 96.8% BPA were obtained. While it was revealed that the reaction proceeded via the methoxide pathway, the reaction over NaAlO_2_ possessed a low *E*_a_ of 75.1 kJ mol^–1^, which is the lowest ever reported so far for heterogeneous catalysis. The catalyst was reusable at least 4 times without severe catalyst deactivation.

## Supporting Information Available

Characterization of SrO and NaAlO_2_ after the reaction; distribution of basic strength via Hammett indicators; estimation of the amount of basic site via titration; procedure for filtration test; characterization of the as-produced BPA; effect of calcination temperature on the catalytic performance of NaAlO_2_; treatment of MeOH with NaAlO_2_ for ^1^H NMR analysis; and reusability test procedure. (PDF)
