# Supplementary material for: Low-Temperature Methanolysis of Polycarbonate over Solid Base Sodium Aluminate
Source: Langmuir. 2024 Feb 26;40(10):5338–47. doi: 10.1021/acs.langmuir.3c03799 (PMC10938891; doi:10.1021/acs.langmuir.3c03799)
Supplement: Supplementary file 1 — la3c03799_si_001.pdf [file la3c03799_si_001.pdf]

Supporting Information

Low temperature methanolysis of polycarbonate over solid base sodium aluminate

Philip Anggo Krisbiantoro[a,b,c], Miyu Sato[d], Tzu-Ming Lin[c], Yu-Chia Chang[c], Tzu-Yun Peng[c], Yun-Chih Wu[c], Weisheng Liao[c], Yuichi Kamiya[e], Ryoichi Otomo[e], Kevin C.-W. Wu*[a,b,c,f,g]

[a] Molecular Science and Technology Program, Taiwan International Graduate Program, Academia Sinica, Taipei 11529, Taiwan

[b] International Graduate Program of Molecular Science and Technology, National Taiwan University, Taipei 10617, Taiwan

[c] Department of Chemical Engineering, College of Engineering, National Taiwan University, Taipei 10617, Taiwan

[d] Graduate School of Environmental Science, Hokkaido University, Nishi 5, Kita 10, Kita-ku Sapporo, 060-0810, Japan

[e] Faculty of Environmental Earth Science, Hokkaido University, Nishi 5, Kita 10, Kita-ku, Sapporo, 060-0810, Japan

[f] Center of Atomic Initiative for New Materials, National Taiwan University, Taipei 10617, Taiwan

[g] Department of Chemical Engineering and Materials Science, Yuan Ze University, Chung-Li, Taoyuan, Taiwan

Corresponding author:

Kevin C.-W. Wu: No. 1, Sec. 4, Roosevelt Road, Taipei, 10617, Taiwan; kevinwu@ntu.edu.tw

Characterization of SrO and $NaAlO_2$ after reaction

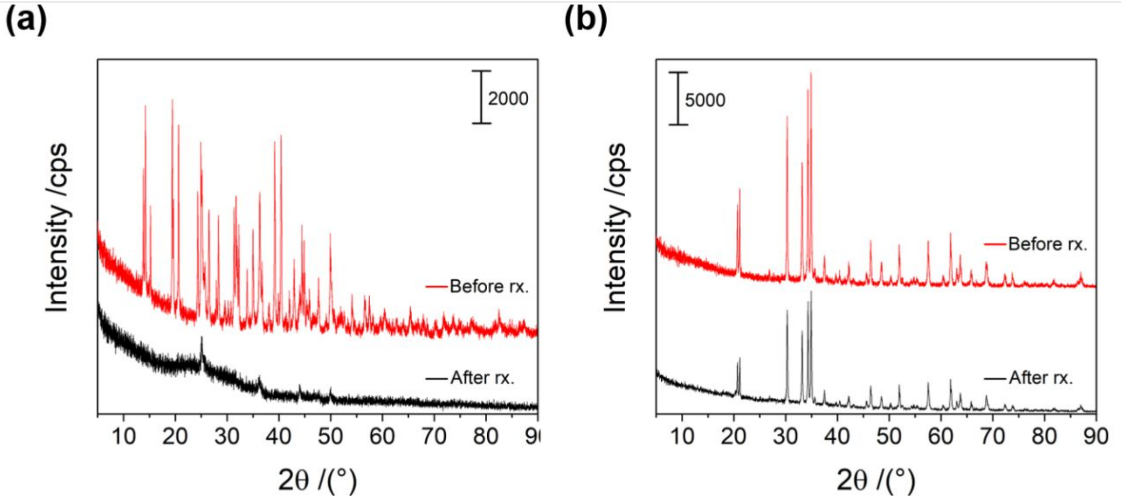

Figure S1. XRD patterns of **(a)** SrO and **(b)** $NaAlO_2$ after reaction. Reaction conditions for PC methanolysis: catalyst weight, 0.2 g; MeOH weight, 5 g; THF weight, 10 g; PC weight, 5 g; reaction temperature, room temperature; and reaction time, 9 h.

Distribution of basic strength via Hammett indicators

To determine the distribution of basic strength via Hammett indicators, four Hammett indicators, e.g., bromothymol blue (H_{-}= 7.2), phenolphthalein (H_{-}= 9.8), alizarin yellow R (H_{-} = 11.0), and 4-nitroaniline (H_{-}=18.4), were used. Typically, 50 mg of catalyst was added into 5 mL of MeOH and 0.1 wt.% of indicator in MeOH (around three drops), followed by sonication for 5 min and separation of the catalyst via filtration before finally visually determining the colour. For bromothymol blue as an indicator, the catalyst that gave a green colour was categorized as a catalyst with $H_{-} \leq 7.2$, while those that gave a light blue or blue colour were considered as a material with $H_{-} \geq 7.2$. For phenolphthalein as an indicator, while pink colour means the catalyst has an H_{-} value of ≥ 9.8, clear colour suggested otherwise. For alizarin yellow R, $H_{-} \geq 11.0$ when it gives light violet colour and $H_{-} \leq 11.0$ when it shows yellow colour. In the case of 4-nitroaniline, the catalyst with $H_{-} \geq 18.4$ should give an orange colour, while a lower value should give a yellowish colour. The detailed results can be seen in **Figure S2**.

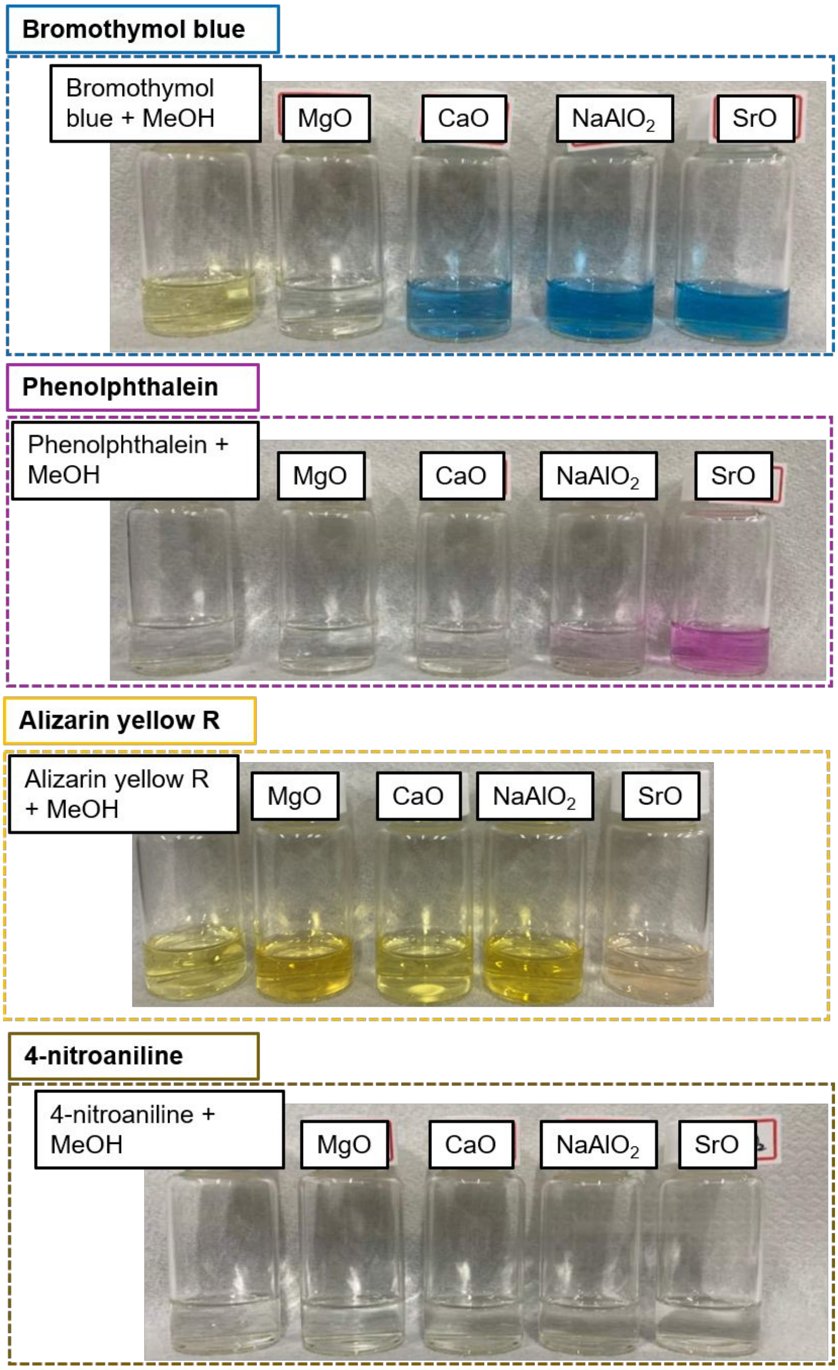

Figure S2. The colour of the solution in the presence of different indicators after MgO, CaO, SrO, or $NaAlO_2$ was previously added and filtered.

Estimation of the amount of basic site via titration

To quantitatively estimate the amount of basic site of the catalyst, a titration technique was applied with bromothymol blue used as an indicator. Typically, 50 mg of catalyst was added into a mixture of 5 mL MeOH and 0.1 wt.% of bromothymol blue in MeOH (around three drops). The mixture was sonicated for 5 min followed by the separation of the solid via filtration. The filtrate was then titrated with 0.05 M of HCl, and the mmol amount of HCl per gram of catalyst was determined as the amount of basic site of the catalyst.

Procedure for filtration test

Typically, 5 g of MeOH and 10 g of THF were placed in a 50 mL round-bottom flask equipped with a magnetic stirrer. PC pellets (5 g) were then added into the flask, which was quickly followed by the addition of 0.2 g of $NaAlO_2$. The mixture was then vigorously stirred for 3 h. Afterward, the catalyst and remaining PC were quickly separated from the filtrate via filtration. While the filtrate was put into a new 50 mL round-bottom flask equipped with a magnetic stirrer, the remaining PC was carefully separated from the catalyst by tweezers, followed by immersion in the fresh MeOH to remove the catalyst from the surface of the PC. The MeOH was then decanted to remove the catalyst. This immersion-decantation experiment was done three times to ensure the thorough removal of the catalyst from the surface of the PC. The PC was then added to the previously separated filtrate, and the mixture was then vigorously stirred for 3 and 6 h to get a reaction with 6 and 9 h reaction times.

Characterization of the as-produced BPA

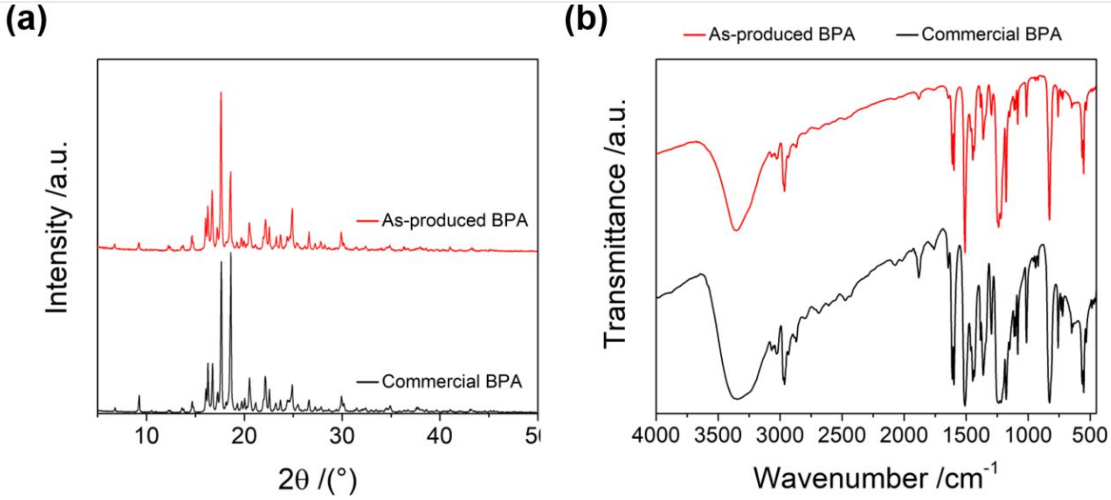

Figure S3. (a) XRD patterns and **(b)** FTIR spectra of as-produced BPA in comparison to the commercially available BPA from Thermo Fisher Scientific.

Effect of calcination temperature on the catalytic performance of $NaAlO_2$

As it is widely reported that the calcination temperature affects the catalytic performance of solid base catalysts, we calcined $NaAlO_2$ at different temperatures and investigated their catalytic performance. A temperature lower than 60 °C, i.e., at 50 °C, was used to clearly see the difference in catalytic performance among catalysts since $NaAlO_2$ has high catalytic performance. As shown in **Figure S4**, without any heat treatment, $NaAlO_2$ gave 71.9% PC conversion and 83.3% BPA yield. When the material was calcined at 200 °C, the conversion and yield increased to 88.9 and 81.1%, respectively. The increase in catalytic performance might be due to a more basic site being generated as the result of the removal of water molecules on the surface of the catalyst.

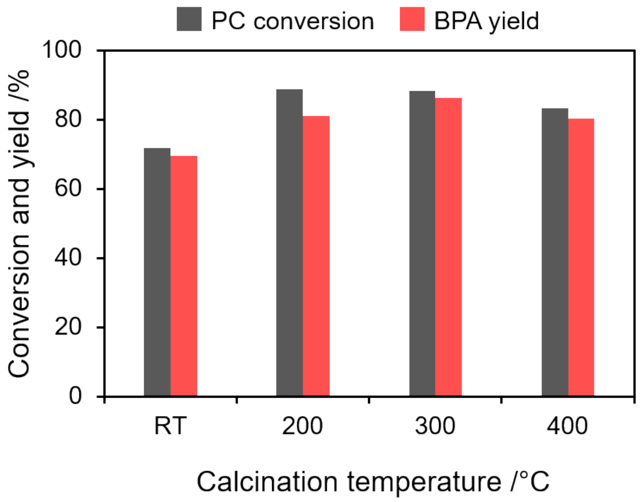

Figure S4. Catalytic performance of $NaAlO_2$ calcined at different temperatures. Reaction conditions: Catalyst, 0.2 g; MeOH, 5 g; THF, 10 g; PC, 5 g; reaction temperature, 50 °C; and reaction time, 1 h.

Treatment of MeOH with $NaAlO_2$ for ^{1}H-NMR analysis

Typically, 5 g $NaAlO_2$ and 0.14 g NaCl were added into 5 g of MeOH, followed by sonication for 10 min and filtration by using a syringe filter. The solvent used for the analysis was DMSO-d_6.

Reusability test procedure

In a typical reusability test experiment, the catalyst after the reaction was washed with THF and MeOH three times each to ensure the thorough removal of the oligomers or monomers from the surface of the catalyst. The catalyst was then dried overnight using a lyophilizer before being reused for the next run of PC methanolysis with THF as a solvent. In the case of < 100% PC conversion, the PC residue was separated from the catalyst during filtration via careful picking by using tweezers, followed by immersion of the picked PC residue in the fresh MeOH to remove the catalyst from the surface of the PC. This immersion-decantation experiment was done three times. The solution from the immersion-decantation was then filtered to collect the catalyst. The catalyst was then dried overnight using a lyophilizer before being reused for the next run of PC methanolysis.
